# Neuronal Signaling Involved in Neuronal Polarization and Growth: Lipid Rafts and Phosphorylation

**DOI:** 10.3389/fnmol.2020.00150

**Published:** 2020-08-14

**Authors:** Michihiro Igarashi, Atsuko Honda, Asami Kawasaki, Motohiro Nozumi

**Affiliations:** Department of Neurochemistry and Molecular Cell Biology, Niigata University School of Medicine and Graduate School of Medical/Dental Sciences, Niigata, Japan

**Keywords:** growth cone, lipid rafts, phosphoproteomics, JNK, super-resolution microscopy, palmitoylation

## Abstract

Neuronal polarization and growth are developmental processes that occur during neuronal cell differentiation. The molecular signaling mechanisms involved in these events in *in vivo* mammalian brain remain unclear. Also, cellular events of the neuronal polarization process within a given neuron are thought to be constituted of many independent intracellular signal transduction pathways (the “tug-of-war” model). However, *in vivo* results suggest that such pathways should be cooperative with one another among a given group of neurons in a region of the brain. Lipid rafts, specific membrane domains with low fluidity, are candidates for the hotspots of such intracellular signaling. Among the signals reported to be involved in polarization, a number are thought to be present or translocated to the lipid rafts in response to extracellular signals. As part of our analysis, we discuss how such novel molecular mechanisms are combined for effective regulation of neuronal polarization and growth, focusing on the significance of the lipid rafts, including results based on recently introduced methods.

## Introduction

Brain development in mammals is believed to involve six steps, including: (1) segmentation of brain regions; (2) neuronal differentiation from neural stem cells; (3) neuronal migration to the appropriate locations; (4) neuronal polarity determination and axon growth as directed by guidance molecules; (5) synaptogenesis; and (6) removal of excess synapses (Sanes et al., 2019). Except for the last step, which depends on neuronal activity, the other steps appear to be regulated by genetic mechanisms. In this review article, we focus on molecular aspects of the fourth step of the above sequence of mammalian brain development.

More than 30 proteins have been characterized based on their involvement in neuronal polarization at the single-cell level (Takano et al., 2019). While many of these proteins likely contribute to neuronal polarization in similar ways, these molecules were discovered in independent studies, and little is known about how these proteins might act in a coordinated fashion. In this review, we focus on the potential role of lipid rafts in neuronal polarization and axon growth (Igarashi, 2019).

## Lipid Rafts: What Is Important for Signaling?

Glycerophospholipids are major components of the plasma membrane, and membrane proteins are incorporated in such lipids (Brown and London, 1998; Lorent and Levental, 2015). According to the classical model, such lipids have high fluidity, behaving like a liquid, due to the unsaturated fatty acids bound to these phospholipids; all of the membrane proteins thus would be flowing in a “sea” of membrane lipids, freely diffusing to anywhere within the membrane. In contrast to such an idea (that the membrane structure is uniform), the concept of the lipid rafts has been postulated. Namely, minor components of the membrane lipids, including cholesterol (sterol) and sphingolipids (sphingomyelin and glycolipids such as gangliosides), are present in a concentrated and clustered form in specific domains of the membrane. Biophysical properties of these minor membrane lipids predict that the lipid raft domain has much lower fluidity than that of the major components (the glycerophospholipids; Lorent and Levental, 2015). These low fluidity regions serve as anchors for specific membrane proteins that reside therein, and the lipid rafts are thought to be “signaling hotspots” for responding to extracellular signals (Lingwood and Simons, 2010; Egawa et al., 2018).

**Figure 1:**
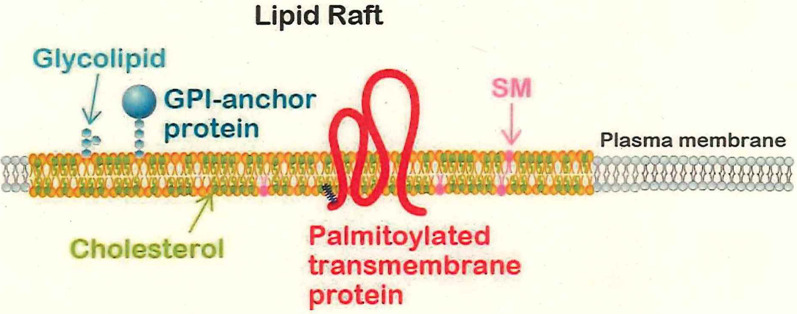
The lipid raft domain. Lipid rafts are composed of sphingolipids such as glycolipids and sphingomyelin (SM), cholesterol, and glycosylated phospholipid (GPI)-anchored or palmitoylated membrane proteins. Lipid rafts are thought to be interspersed among non-raft domains that are composed of the glycerophospholipids and exhibit high fluidity. The lower fluidity of the lipid rafts is presumed to lead to retention and localized concentration of membrane proteins that participate in signal transduction in response to extracellular signals.

Two types of membrane proteins are thought to be specifically associated with lipid rafts: glycosylated phosphatidylinositol (GPI)-anchored proteins (Saha et al., 2016) and palmitoylated proteins. GPI-anchored proteins are located at the cell surface and are attached to the plasma membrane with a GPI anchor, but cannot directly interact with intracellular signaling proteins; thus, GPI-anchored proteins require co-receptors that possess transmembrane domains. GPI-anchoring sugar chains are synthesized in the endoplasmic reticulum (ER) and then undergo fatty acid modification at PI within the Golgi apparatus before being sorted to the plasma membrane (Saha et al., 2016). The resulting GPI-anchored proteins have been observed to repeatedly undergo rapid gathering and scattering within lipid rafts (Suzuki et al., 2017; Figure 1).

Protein palmitoylation is an S-acylation modification of clustered cysteine residues; this protein modification is performed in the Golgi apparatus (Chini and Parenti, 2009; Resh, 2016). For soluble proteins, palmitoylation simply endows the targets with an affinity for the plasma membrane; for transmembrane proteins, palmitoylation is believed to direct the targets for sorting to the lipid raft domains (Stepanek et al., 2014; Lorent and Levental, 2015). More than 20 palmitoyltransferases have been identified in mammals, and each of these enzymes is thought to have specific physiological substrates (Fukata et al., 2016). Of the major palmitoylated proteins in the adult brain, more than half are transmembrane proteins (Kang et al., 2008). Previous work has demonstrated the significance of palmitoylated transmembrane proteins in neurobiology (Vallejo et al., 2017; Hayashi, 2020); the relationship of palmitoylation to neuronal polarity will be addressed later in this review article.

## Neuronal Polarization and the Need for Its Rapid Determination

The selection of the specific regions of a neuron where growth cones form is an important problem; neuronal polarity is key to the formation of the axon (a single output process) and dendrites (multiple input processes; Laumonnerie and Solecki, 2018). Neuronal polarity determination has been classified into five stages (Dotti et al., 1988), namely, stage 1: initiation of the emergence of the minor process(es); stage 2: the growth of the minor processes; stage 3: axon specification; stage 4: dendritic specification; and stage 5: synaptogenesis. Among these steps, the transition from stage 2 to stage 3 has been the most intensively studied (Funahashi et al., 2020). Most of the previous studies on the establishment of neuronal polarity have examined cell-autonomous signaling pathways in individual (single) cells in *in vitro* culture systems (Funahashi et al., 2020). Based on these previous studies, a tug-of-war model (Lalli, 2014) has been adopted to explain neuronal polarization. This model (Figure 1) is based on the experimental facts that although each neuron in dissociation culture (particularly when grown on artificial culture substrates) has an intrinsic mechanism for neuronal polarization, at stage 2, each minor neuronal process performs the inter-dependent interactions for signaling in a tug-of-war. After spending a relatively long time (~48 h) at stage 2, the model explains that the sole process that “wins” this “tug of war” requires rapid growth to differentiate successfully into an axon at stage 3 (Lalli, 2014; Guo and Cheng, 2015).

**Figure 2:**
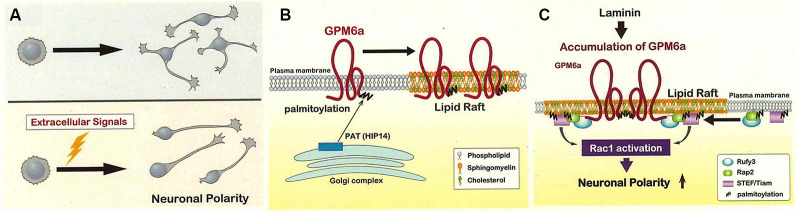
Lipid rafts may be the site of signaling for the determination of the neuronal polarity.** (A)** The polarity of each neuron is probably not determined in an inconsistent way (*upper*) but instead is synchronized *in vivo* by extracellular signals exchanged among neurons (*lower*). The *lower* mechanism is expected to shorten the time for polarity determination compared to the *upper* one. **(B)** Glycoprotein M6a (GPM6a) is palmitoylated and sorted to the lipid rafts in the neuronal plasma membrane. GPM6a is believed to be palmitoylated at cysteine clusters (located near the protein’s *N*-terminus) *via* a reaction catalyzed by the HIP14 or ZHHC17 palmitoyltransferases; modification would occur within the Golgi apparatus, and GPM6a then would be inserted into the lipid raft domains (Butland et al., 2014). Although the non-palmitoylated form of GPM6a is localized to non-raft domains of the plasma membrane, this form of GPM6a does not appear to mediate biological effects in response to extracellular signals (Honda et al., 2017a). **(C)** Laminin induces the assembly of signaling molecules downstream of GPM6a around lipid rafts, an event that contributes to the rapid determination of polarity (see Honda et al., 2017a). GPM6a, Rap2, and Tiam2 are present in the lipid rafts with Rufy3, an adaptor protein that acts as a linker between GPM6a and Rap2-Tiam2 (Honda et al., 2017a,b). Tiam2 is a guanine nucleotide exchange factor (GEF) that activates Rac and is expected to contribute to the rapid determination of polarity. Modified from Honda et al. (2017a).

However, it seems unlikely that the signaling leading to polarization of neurons occurs spontaneously under *in vivo* conditions (Namba et al., 2014). For *in vivo* mammalian brain development, each neuron within a group would have to acquire polarity simultaneously, and then also grow an axon simultaneously in the same direction, a series of events that seems far more complicated than the simple tug-of-war mechanism. Namely, *in vivo*, stage 2 (a stage of undecided polarity) cannot persist for an extended interval, and the transition from stage 2 to stage 3 (a stage of defined polarity) cannot proceed in a disorderly fashion. It is difficult to imagine that intrinsic factors alone would be expressed *in vivo* in a large number of the neurons just before stage 3 in a manner that would permit (despite the restricted time course) synchronization of the polarization with the axon growth direction (Namba et al., 2014). The mechanisms of stage 3 itself (rapid axon growth) sometimes appear to conflict with those proposed for the transition from stage 2 to stage 3 (Takano et al., 2019; Figure 2A).

Thus, there appears to be a role for extrinsic factors in inducing polarity determination within the neuronal population. Candidates for such signals have been identified (Takano et al., 2019), and include extracellular matrix components such as laminin (LN), a protein that is highly abundant in the developing brain (Esch et al., 1999; Randlett et al., 2011; Johnson et al., 2012; Honda et al., 2017a,b; Serjanov et al., 2018). LN facilitates neuronal polarity determination, as demonstrated by the ability of exogenously supplied LN to permit neurons to “skip” stage 2 of development (Honda et al., 2017a,b).

## Neuronal Polarization Related to Lipid Rafts

### Signaling Molecules for Polarization in Lipid Rafts

Among the many proteins involved in neuronal polarization (Takano et al., 2019), more than 10 species that are present upstream of the signaling have been reported to be present in lipid rafts or to be translocated to lipid rafts when the corresponding signals are activated (Table 1). These results suggest that those molecules are likely to function in polarization signaling as the concentrated forms in lipid rafts.

**Table 1:** Proteins reported to localize to lipid rafts or to be translocated to lipid rats in response to extracellular stimuli.

**A. Receptors and cell adhesion molecules**
TrkB (Assaife-Lopes et al., 2010; Mandyam et al., 2017)
IGF-1R (Sural-Fehr et al., 2019)
Neuropilin/Plexin complex (Dang et al., 2012)
Integrin (Decker et al., 2004)
Thy-1 (Ledesma et al., 1998)
**B. Protein kinases**
CaMKI (Davare et al., 2009)
Glycogen synthase kinase-3 (Sui et al., 2006)
SAD-B (Rodríguez-Asiain et al., 2011)
Akt (Bryant et al., 2009)
Fyn (Ko et al., 2005)
**C. Other intracellular signaling molecules**
PI3K (Zheng et al., 2014)
Wnt-Dvl (Frizzled; Haack et al., 2015)
Ras/Rap (Zhang et al., 2018)
Rac1 (Fujitani et al., 2005; Grider et al., 2009; Köster et al., 2014; Lee et al., 2016)
V-ATPase (Kanda et al., 2013; Makdissy et al., 2018)

*The proteins involved in polarization are listed above*.

Neuronal polarization is known to depend on the positioning of the Golgi apparatus, and thus, the biochemical mechanisms in that organelle should have important effects on this event (Villarroel-Campos et al., 2016; Tortosa and Hoogenraad, 2018; Caracci et al., 2019). Although such biochemical processes are not completely understood, one essential modification performed in the Golgi apparatus is protein palmitoylation, which regulates the trafficking of proteins for axon specification (Rodríguez-Asiain et al., 2011; Tortosa et al., 2017; Tortosa and Hoogenraad, 2018). In mammals, protein palmitoyltransferases (PATs, the enzymes responsible for this reaction) are concentrated in the cis-Golgi and catalyze S-palmitoyl acylation of cysteine residues in target proteins (Ernst et al., 2019). Such fatty acylation of soluble proteins is believed to recruit these proteins to the plasma membrane. This modification also is employed for membrane proteins, although the purpose of palmitoylation of such proteins (which are already membrane-associated) is less apparent. Notably, however, in various cells (including the neuron), palmitoylation increases recruitment of such transmembrane proteins to lipid rafts (Linder and Deschenes, 2007; Hayashi, 2020). Palmitoylation is thought to modify the membrane trafficking of the target proteins, possibly by changing the curvature of the sorting vesicles carrying these proteins (Ernst et al., 2019).

In the adult rodent brain, more than 20 species of major palmitoylated proteins have been identified; more than half are transmembrane proteins, a class that includes Glycoprotein M6a (GPM6a; Kang et al., 2008).

### GPM6a Signaling in Response to LN

GPM6a, a potential regulator of neuronal growth, is a major membrane protein of the growth cone (Nozumi et al., 2009); specifically, GPM6a is a four-transmembrane-domain protein that is known to be highly expressed in differentiated neurons (Möbius et al., 2008). This gene product is a major palmitoylated protein in the adult brain (Kang et al., 2008). Although GPM6a’s exact roles remained unclear, we suspected that this protein might be a signal transducer for LN-dependent signaling. Notably, inhibition of GPM6a palmitoylation abolished LN-dependent determination, indicating that the trafficking of this protein to lipid rafts is essential to GPM6a’s mechanism of action (Honda et al., 2017a,b), even though GPM6a, being an intrinsic membrane protein already localizes to the plasma membrane (Ito et al., 2018).

Using proteomics, a GPM6a-Rufy3-Rap2a-Tiam2 complex was identified in lipid rafts (Honda et al., 2017a). Rufy3 (also called Singar 1; Mori et al., 2007) and Tiam2/STEF both are known to be involved in neuronal polarization. Tiam2, a Rac guanine nucleotide exchange factor (GEF), determines the site of axon extension *via* the rapid accumulation of the GTP-bound form of Rac1 (Nishimura et al., 2005). This accumulation of GTP-Rac1 may be useful for organizing multiple otherwise-unrelated signaling molecules that contribute to polarization. For example, the activation of Rac1 by positive feedback *in vivo* is probably essential to speedy polarization (Acevedo and González-Billault, 2018; Dupraz et al., 2019; Takano et al., 2019); proximity to members of the Tiam family (proteins that serve as Rac GEFs) would facilitate this process. It is physiologically conceivable that Rap2 (Bruurs and Bos, 2014), a member of the Ras GTPase family that is highly palmitoylated (Uechi et al., 2009; Baumgart et al., 2010), is present in lipid rafts, such that the presence of activated Tiam2 in the lipid rafts contributes to polarization (Honda et al., 2017a,b).

Rufy3 is (in *in vitro* experiments) a multiple adapter protein for small GTPases (Fukuda et al., 2011) and has been shown to bind to activated Rap2 (Kukimoto-Niino et al., 2006; Honda et al., 2017a,b). Rufy3 also is involved in neuronal polarity (Mori et al., 2007), for which the only identified related signaling molecule was PI-3-kinase (PI3K; Mori et al., 2007). In *in vivo* signaling, lipid rafts may connect GPM6a to Rap2-Tiam *via* Rufy3; indeed, GPM6a can induce the translocation of Rufy3 to lipid rafts (Honda et al., 2017a,b).

### Human Neuropsychiatric Diseases and Polarization

GPM6a is known to be a good endogenous substrate of HIP14/Zdhhc17, a palmitoyl acetyltransferase (protein palmitoyl acyltransferase; PAT) implicated in Huntington disease, a human hereditary neurodegenerative disease (Butland et al., 2014; Figure 2B).

Also, GPM6a, Rufy3, Rap2, and Tiam2 (Figure 2C) all have been implicated in studies of important psychiatric diseases, including analyses of human patient neuropathologies and murine models (Funk et al., 2012; Bhattacherjee et al., 2017; Ma et al., 2018; Aberg et al., 2020). Notably, genome-wide association study (GWAS) identified the genes encoding GPM6a and Rufy3 as loci associated with an elevated risk of human schizophrenia and depression, respectively (Ma et al., 2018; Aberg et al., 2020). Since these diseases are thought to be partly due to the genetic lability of some genes in brain development, such results suggest that GPM6a and downstream molecules have physiological roles in the development of neurons and that GPM6a in lipid rafts may be involved in a key step of neuronal morphogenesis.

## Membrane Recycling Mechanisms in Lipid Rafts; Newly Observed Using Super-Resolution Microscopy

### Technical Merits and Power of Super-Resolution Microscopy for Analysis of Membrane Trafficking

To better understand the role of membrane trafficking in axonal growth, the precise relationship between both cytoskeletal and membrane components must be clarified. Live imaging has greatly contributed to the understanding of such mechanisms (Igarashi et al., 1996; Tamada and Igarashi, 2017; Dubey et al., 2018; Meka et al., 2019). However, live imaging of growing axons has remained a challenge: the vesicles and cytoskeleton in the growth cone are highly crowded, meaning that each labeled structure overlaps with others, impeding discrimination among the various components. Additionally, for *conventional* confocal microscopy, the diffraction limit of optical microscopy (~200 nm) has precluded precise analyses of vesicles and cytoskeletal structures in growth cones (Igarashi et al., 2018; Schermelleh et al., 2019).

Recently, several types of super-resolution microscopy have been developed (Hauser et al., 2017; Igarashi et al., 2018; Schermelleh et al., 2019). These methods employ fluorescence microscopy devices to observe intracellular molecules, permitting researchers to overcome the optical diffraction limit and achieve resolutions of 50–100 nm. These new techniques not only make it possible to observe smaller objects but also facilitate the analysis of densely distributed materials such as vesicles and cytoskeletal components in the growth cone (Nozumi and Igarashi, 2018). Also, super-resolution microscopy provides three-dimensional images and so is superior to confocal microscopy in this context (Igarashi et al., 2018).

One super-resolution technique, structured illumination microscopy (SIM), can visualize the fine structure of cells by calculating the interference (moiré) patterns induced by irradiation with striped-pattern excitation light (Gustafsson, 2008). Using SIM, lateral and axial dimensions of approximately 100 and 300 nm (respectively) can be visualized, making super-resolution microscopy useful for tracking molecular dynamics and movements in live-cell imaging (Demmerle et al., 2017; Richter et al., 2018).

### Membrane Recycling in Lipid Rafts Contributes to Axon Growth

Although biochemical evidence for the existence of the lipid rafts accumulated until 2010, the idea of the lipid rafts remained a hypothesis. This challenge remained because the visualization of the lipid raft domains remained impossible up to that time. However, the development of super-resolution microscopy permitted the observed lipid rafts in various cell types, leading to the wider acceptance of this concept (Owen et al., 2012). There are several styles of super-resolution microscopy that use distinct probes; each of these methods has successfully permitted the visualization of lipid rafts (Tobin et al., 2014; Chen et al., 2015; Hartley et al., 2015; Stahley et al., 2016; Gao et al., 2017; Schlegel et al., 2019; Angelopoulou et al., 2020). These new results have contributed to models suggesting possible roles for lipid rafts in multiple cellular pathways (Raghunathan and Kenworthy, 2018).

In the neuron, however, such an approach had not been applied, given the elevated density of cholesterol and sphingolipids, particularly gangliosides, in neural membranes. Only recently has the development of 3D-SIM-type super-resolution microscopy permitted imaging of the dynamic endocytotic processes of the lipid raft domains in the growth cone (Nozumi et al., 2017).

**Figure 3:**
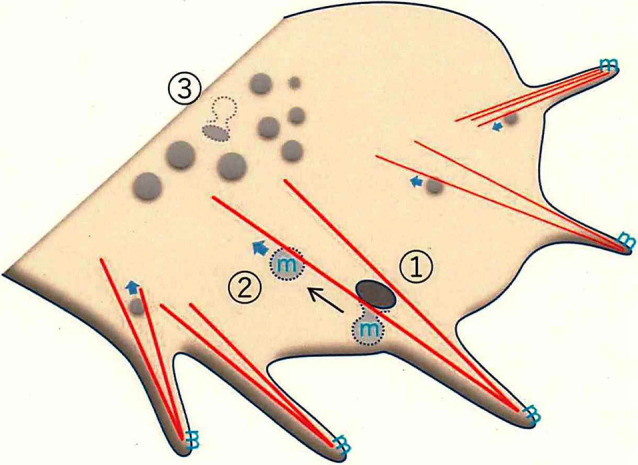
Membrane trafficking in the growth cone, as revealed by super-resolution microscopy. F-actin-dependent endocytosis occurs in the peripheral (P-) domain of the growth cone. The leading edge protrudes as filopodia, which have dense F-actin bundles (*red* lines). F-actin-bundling for filopodial formation induces endorphin-mediated endocytosis (EME; ①; Nozumi et al., 2017). EME depends on F-actin located in the Z-axis direction (see Igarashi et al., 2018). GPM6a (the symbol “m” in *blue*), distributed in the lipid rafts, is endocytosed through EME. The EME-dependent vesicles move in a retrograde direction to the central (C-) domain of the growth cone (②). Classical clathrin-mediated endocytosis (CME) mainly occurs at the bottom of the growth cone membrane (GCM; ③).

3D-SIM depends on the use of D4 (a molecule derived from bacterial theta toxin that shows specific binding to membrane cholesterol); a fusion of green fluorescent protein (GFP) to D4 (GFP-D4) can be used as a probe for labeling cholesterol (Ohno-Iwashita et al., 2004; Ishitsuka et al., 2011). By combining this probe and super-resolution microscopy, we succeeded in visualizing neuronal membrane lipid rafts (Nozumi et al., 2017). These lipid rafts showed movements similar to those seen for GPM6a itself and clathrin-independent endocytosis at the leading edge. Thus, we infer that the lipid rafts are associated with F-actin bundling at the leading edge, where these structures undergo highly dynamic movements as part of axonal growth (Figure 3).

Several studies revealed that the impaired endocytosis of the lipid raft domains induced abnormal neuronal signaling, suggesting that lipid rafts are critical for endocytotic signaling pathways (Laudati et al., 2016; Nogueira-Rodrigues et al., 2016). Biochemically, these signaling events were thought to be clathrin-dependent (Qiu et al., 2011); however, super-resolution live-imaging of GPM6a- and cholesterol-dependent endocytosis in the growth cone revealed that these events were clathrin-independent (Nozumi et al., 2017) and dynamin and endophilin dependent. Dynamin is a GTPase that contributes to membrane cleavage and endocytosis (De Camilli et al., 1995). Endophilin is a BAR-domain protein that regulates membrane curvature (Kjaerulff et al., 2011; Gallop, 2020). The characteristics of these endocytotic events somewhat resemble “fast (or ultrafast) endocytosis,” a process seen at presynaptic terminals (Watanabe et al., 2014, 2018; Wu et al., 2014; Boucrot et al., 2015; Renard et al., 2015; Watanabe and Boucrot, 2017; Milosevic, 2018).

## Phosphorylation at Stage 3 for Axon Growth

At stage 3, lipid rafts are thought to still be involved in axon formation *via* signaling in response to axon guidance molecules, including events such as protein phosphorylation (Guirland et al., 2004; Hérincs et al., 2005; Kamiguchi, 2006). It has been reported that cholesterol is more enriched in the growth cone at early stages than at later stages (Chauhan et al., 2020), suggesting the importance of the lipid rafts in this process. GAP-43 (growth-associated protein of 43-kDa), a neuronal growth-associated membrane protein also is known to be a lipid raft resident in the developing brain (Denny, 2006; Tong et al., 2008; Sekino-Suzuki et al., 2013; Kalinowska et al., 2015; Forsova and Zakharov, 2016). Recently, using phosphoproteomics of the growth cone, the most frequently phosphorylated site (among all of the identified growth cone membrane (GCM) proteins) was identified as S96 of GAP-43 (Kawasaki et al., 2018). The responsible kinase was identified as JNK, an enzyme whose activity also is dependent upon signaling in lipid rafts (Makdissy et al., 2018). Originally, JNK was postulated to be the transducer of apoptotic signals in multiple cell types (Hibi et al., 1993; Bogoyevitch et al., 2010); in neurons, this pathway was shown to induce axon degeneration (Shin et al., 2012). Three isoforms of the kinase (JNK1, 2, and 3) have been long been known to be related to cell death; recently, however, there is accumulating evidence that JNK has positive roles in neuronal development in the brain (Waetzig et al., 2006; Tararuk et al., 2006), including neurogenesis (Amura et al., 2005; Xu et al., 2014; Lim et al., 2015), neuronal migration (Kawauchi et al., 2003; Westerlund et al., 2011; Myers et al., 2014, 2020; Kawauchi, 2015), polarization (Slater et al., 2013), and axon growth and guidance (Oliva et al., 2006; Shafer et al., 2011; Feltrin et al., 2012; Qu et al., 2013; Sun et al., 2013). As has been hypothesized for other kinases (e.g., PKA, Akt, GSKβ, Cdk5, and Rho), JNK activation and phosphorylation of other substrates (Kawasaki et al., 2018; Ishikawa et al., 2019) is physiologically necessary for axon growth in the developing brain (Yamasaki et al., 2011). Protein phosphorylation is an important regulatory mechanism in cell development and homeostasis (Humphrey et al., 2015). At stage 3, rapid axon growth requires a signaling trigger, and protein phosphorylation is the most likely mediator of such a trigger.

Pin1 is a member of the peptidyl-prolyl isomerases (PPIases), a class of proteins that bind phosphorylated S/T-P motifs and catalyze the cis/trans-isomerization of P-containing peptides. This reaction switches the conformation and thereby the function(s) of the substrate proteins, including activity, protein-protein interaction, stability, and subcellular localization (Yaffe et al., 1997; Lu et al., 1999; Park et al., 2012; Litchfield et al., 2015). Pin1 is enriched in the brain and has been shown (by proteomics) to be present in the growth cone (Nozumi et al., 2009; Estrada-Bernal et al., 2012; Igarashi, 2014; Chauhan et al., 2020). The protein is known to activate protein kinases that participate in phosphorylation cascades (Litchfield et al., 2015). Indeed, JNK is directly kept activated by Pin1 (Park et al., 2012; Litchfield et al., 2015), suggesting that JNK activation *via* Pin1 likely occurs in the developing neuron and the growth cone from stages 1 to 3.

**Figure 4:**
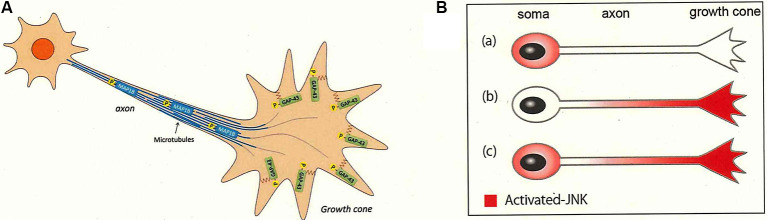
JNK activity in the axon and its substrates for axonal growth. JNK is activated in the developing neurons (Hirai et al., 2011; Yamasaki et al., 2011; Coffey, 2014). **(A)** JNK-dependent substrates are sorted to the distal axon and the growth cone. Phosphorylated segments of GAP-43 (peptides pS96 and pT172) and MAP1B (peptides pS25 and pS1201) are sorted to the plasma membrane and the microtubules in the growth cone of the distal axon, respectively. These substrate proteins are phosphorylated by JNK in the cell bodies before undergoing anterograde axonal transport or are phosphorylated by JNK proximal to the growth cone area [see **(A)**]. See Kawasaki et al. (2018) and Ishikawa et al. (2019). **(B)** JNK may be distributed within the growing axons in one of three patterns: (Ba) only in the cell bodies, (Bb) only in the growth cone, or (Bc) in the whole neuron. Our experimental results indicate that (Ba) or (Bc) are more likely (Kawasaki et al., 2018).

Recent advances have yielded phosphoproteomics, a powerful method for comprehensive and quantitative identification of the *in vivo* phosphorylation sites used in a given system (von Stechow et al., 2015; Invergo and Beltrao, 2018). Our application of this technique to GCM proteins (Ellis et al., 1985) led to the identification of more than 30,000 phosphopeptides representing ~4,600 different phosphorylation sites in ~1,200 proteins (Kawasaki et al., 2018; Igarashi et al., 2020). The phosphorylation of several frequently phosphorylated sites, including the S96 and T172 peptides of GAP-43 and the S25 and S1201 peptides of microtubule-associated protein 1B (MAP1B), is JNK dependent (Kawasaki et al., 2018; Ishikawa et al., 2019; Figure 4A). GAP-43 and MAP1B are classical axon growth markers and are highly phosphorylated (Skene, 1989; Riederer, 2007; Holahan, 2017). However, a subset of these frequently phosphorylated sites [e.g., peptide S41 of GAP-43, which is phosphorylated by PKC (Denny, 2006)] were not detected by phosphoproteomic analysis of the GCM. It remains unclear whether JNK is activated only in the cell bodies of the developing neuron, or in the axons or the growth cone at stage 3. If activation occurs in the cell bodies, JNK-phosphorylated substrates would have to undergo anterograde axonal transport to the growth cone; alternatively, if activation occurs in the axon or growth cone, JNK would catalyze local modification of substrates (Figure 4B).

Selected groups of *C. elegans* neurons have been found to require JNK and its upstream kinase (DLK, also referred to as MAP3K) for the regeneration of their axons (see review by Shimizu and Hisamoto, 2020); notably, a lack of JNK resulted in abnormal axonal growth (Tank et al., 2011). Thus, elevated JNK activity appears to be needed for axon maintenance in a wide range of organisms. However, the proteins located downstream of JNK in the *C. elegans* pathway (Chen et al., 2011) appeared to be totally different from the highly phosphorylated substrates identified in our phosphoproteomic analysis, and the JNK-dependent phosphorylated sites, which were analyzed using bioinformatic tools, appeared to be conserved only within components of the analogous vertebrate pathway (Igarashi and Okuda, 2019). Thus, while the need for JNK activity in axon growth/regeneration is conserved between model invertebrates and the mammalian central nervous system, JNK kinase appears to target distinct substrates and phosphorylation sites in these systems (Igarashi and Okuda, 2019).

## Conclusions

In the context of mammalian neuronal polarization based on membrane trafficking, the molecular characterization of lipid rafts based on current detergent-resistant membrane fractions may not be sufficient to understand the hotspots of neuronal signaling. One of the new techniques addressing this issue is enzyme-mediated activation of the radical source (EMARS), which provides specific labeling of lipid rafts (Kotani et al., 2018). Given that only small amounts of proteins are collected after EMARS labeling, this method is still under development; nonetheless, the efficient nature of this labeling procedure holds promise for further expansion of its application.

We may re-examine the signaling pathways for polarity determination in neurons, each of which was previously examined by independent experiments. Portions of these pathways may be related to each other, and others may be proceeded independently and in parallel. Some of the earliest experiments are currently thought to be inappropriate for determining RNAi specificity, or for application to *in vivo* neuronal development of the brain. Also, in retrospect, several of the earlier experiments in this field would not have been able to discriminate effects in neuronal polarization from those in rapid axon growth. As mentioned above, the tug-of-war model developed based on *in vitro* results cannot simply be extended to *in vivo* polarization, given that neurons *in vivo* need to initiate growth in a synchronized fashion and to extend their axons in a single consistent direction.

Such re-examination also may require new methods. For example, phosphoproteomic analysis and super-resolution microscopy are expected to be powerful tools for characterizing the role of trafficking mechanisms in neuronal polarization. Although neuronal polarization and axonal growth appear to be relatively simple, understanding of these events has been more of a challenge than understanding other developmental stages; this difference may reflect the fact that considerably larger numbers of the proteins are involved in these processes, as proteomic and phosphoproteomic analyses have revealed.

## Author Contributions

AH, AK, and MN produced the figures based upon their own original articles. MI wrote the article.

## Conflict of Interest

The authors declare that the research was conducted in the absence of any commercial or financial relationships that could be construed as a potential conflict of interest.
